# *Yersinia enterocolitica* Serum Resistance Proteins YadA and Ail Bind the Complement Regulator C4b-Binding Protein

**DOI:** 10.1371/journal.ppat.1000140

**Published:** 2008-08-29

**Authors:** Vesa Kirjavainen, Hanna Jarva, Marta Biedzka-Sarek, Anna M. Blom, Mikael Skurnik, Seppo Meri

**Affiliations:** 1 Department of Bacteriology and Immunology, Haartman Institute, University of Helsinki, Helsinki, Finland; 2 Helsinki University Central Hospital Laboratory Diagnostics, Helsinki, Finland; 3 Department of Laboratory Medicine, Malmö University Hospital, University of Lund, Malmö, Sweden; Tufts University School of Medicine, United States of America

## Abstract

Many pathogens are equipped with factors providing resistance against the bactericidal action of complement. *Yersinia enterocolitica*, a Gram-negative enteric pathogen with invasive properties, efficiently resists the deleterious action of human complement. The major *Y. enterocolitica* serum resistance determinants include outer membrane proteins YadA and Ail. Lipopolysaccharide (LPS) O-antigen (O-ag) and outer core (OC) do not contribute directly to complement resistance. The aim of this study was to analyze a possible mechanism whereby *Y. enterocolitica* could inhibit the antibody-mediated classical pathway of complement activation. We show that *Y. enterocolitica* serotypes O:3, O:8, and O:9 bind C4b-binding protein (C4bp), an inhibitor of both the classical and lectin pathways of complement. To identify the C4bp receptors on *Y. enterocolitica* serotype O:3 surface, a set of mutants expressing YadA, Ail, O-ag, and OC in different combinations was tested for the ability to bind C4bp. The studies showed that both YadA and Ail acted as C4bp receptors. Ail-mediated C4bp binding, however, was blocked by the O-ag and OC, and could be observed only with mutants lacking these LPS structures. C4bp bound to *Y. enterocolitica* was functionally active and participated in the factor I-mediated degradation of C4b. These findings show that *Y. enterocolitica* uses two proteins, YadA and Ail, to bind C4bp. Binding of C4bp could help *Y. enterocolitica* to evade complement-mediated clearance in the human host.

## Author Summary

To cause disease in humans, pathogenic bacteria have to evade the versatile immune system of the host. An important part of innate immunity is the complement system that is composed of over 30 proteins on host cells and in blood able to detect and destroy foreign material. To survive, bacteria can bind complement regulator proteins onto their surfaces and thus inhibit the activation of complement. Previously, it has been shown that food-borne diarrhoea-causing *Yersinia enterocolitica* can survive in human serum because of two bacterial surface proteins, YadA and Ail. These proteins have been shown to bind a complement alternative pathway regulator, factor H. Here, we show that both proteins also bind the classical and lectin pathway inhibitor, C4b-binding protein. These results together explain the serum resistance of *Y. enterocolitica*. The ability to evade complement attack is apparently important for the pathogenicity of *Yersinia enterocolitica*.

## Introduction

*Yersinia enterocolitica* is a food-borne enteric pathogen of humans and animals. The invasive strains predominantly belong to serotypes O:3, O:9, O:5,27 and O:8. Successful colonization of the intestinal tract is the prerequisite for *Y. enterocolitica* infection. Bacteria pass through the acidic content of the stomach and reach the small intestine. Soon after invading the M cells, bacteria enter the lamina propria and encounter innate and adaptive immune responses [1]. *Y. enterocolitica* can cause enterocolitis, mesenteric lymphadenitis and, as a post-infectious complication, reactive arthritis [2]. Typically, infections lead to specific antibody responses. Mechanisms whereby *Y. enterocolitica* can evade the immune system killing and why it causes reactive arthritis are not fully understood.

The complement system is a crucial constituent of the innate immunity. Its activation via the classical (CP), lectin (LP) or alternative pathway (AP) may lead to the killing of microbes by direct lysis or by complement opsonin-enhanced phagocytosis [3]. Many pathogens, however, can resist complement attack [4]. Under microbe-free circumstances activation of the complement system must be effectively controlled as excessive activity could cause complement consumption, host cell damage or inflammation. C4b-binding protein (C4bp) down-regulates complement activity by acting as a fluid-phase inhibitor of the CP or LP [5]. The predominant form of this large (570 kDa) octopus-shaped glycoprotein [6] consists of seven identical α-chains and one β-chain [7],[8]. The α- and β-chains are composed of eight and three complement control protein (CCP) domains each, respectively. The chains are bundled together by disulphide bonds at their most C-terminal parts [9]. C4bp inhibits CP and LP activation at steps that involve C4b. The N-terminal domains of the C4bp α-chains bind C4b to prevent the assembly of the CP C3-convertase (C4b2a), accelerate its natural decay and render C4b susceptible for factor I (FI) -mediated cleavage and inactivation [10].

Distinct pathogenic microorganisms have been demonstrated to be able to bind host complement regulators, such as factor H (FH) or C4bp, to exploit their protective properties and prevent complement activation on the microbial surfaces. These pathogens include e.g. *Streptococcus pyogenes*, *S. pneumoniae*, *Neisseria gonorrhoeae*, *N. meningitidis*, *Borrelia burgdorferi*, *Escherichia coli* K1, *Moraxella catarrhalis*, *Bordetella pertussis*, *Haemophilus influenzae* and *Candida albicans* [11]–[25].

*Y. enterocolitica* resists efficiently complement-mediated killing [26]–[29]. This resistance depends mainly on two outer membrane proteins YadA and Ail [29]–[33], both expressed exclusively at 37°C. YadA, encoded by the virulence plasmid (pYV), is a trimeric (monomer 43–45 kDa), lollipop-shaped protein composed of the head, neck, coiled-coil stalk and membrane anchor domains. The trimer projects 30 nm out from the outer membrane to form a fibrillar matrix covering the bacterial surface [34],[35].

Ail is a 17 kDa protein encoded chromosomally. It is predicted to comprise eight membrane spanning β-strands and four extracellular loops located close to the cell membrane [36],[37]. Ail seems to be masked to some extent by the distal parts, O-antigen (O-ag) and the outer core (OC), of lipopolysaccharide (LPS) [30]. Unlike in many other Gram-negative bacteria, in *Y. enterocolitica* serotype O:3, the O-ag homopolymer and the OC hexasaccharide are linked to the inner core forming a branched structure. Though both O-ag and OC are needed for colonization of the gut [38],[39] their role in serum resistance appears to be indirect [30].

Similar to other pathogens also *Y. enterocolitica* binds the AP inhibitor FH [40] and we recently demonstrated that *Y. enterocolitica* serotype O:3 bacteria in fact use both YadA and Ail to bind the AP regulator FH (Biedzka-Sarek et al., submitted for publication) but no studies on possible regulation of the CP or C4bp binding to the bacterium have been reported. Since *Y. enterocolitica* efficiently escapes all the complement activation pathways we examined in this study whether *Y. enterocolitica* also interacts with the major CP and LP regulator, C4bp. We show that C4bp binding to *Y. enterocolitica* is also mediated by YadA and Ail. Since the proteins are located at different layers on the bacterial surface, Ail is masked by O-ag and OC while YadA is well surface-exposed. In consequence, Ail binds C4bp when not blocked by O-ag and OC, while YadA-mediated C4bp-binding occurs regardless of the LPS expression status. As an end result the *Y. enterocolitica* –bound C4bp retains its function thereby modulating CP and LP activation on the bacterial surface.

## Materials and Methods

### Human serum, proteins and reagents

Human serum samples devoid of anti-*Yersinia* antibodies were collected from healthy human donors and stored at −70°C. Serum was heat-inactivated (HIS) by incubation for 30 min at 56°C. C4bp with protein S was purified from pooled human plasma as described previously [41]. C4b, factor I and factor H were supplied by Calbiochem. C4bp was labeled with ^125^I (NEN, Boston, MA) using the Iodogen method [42]. Triton X-114 (Tx-114) and heparin sodium salt were purchased from Sigma Chemicals. Phosphate-buffered saline (PBS), Veronal-buffered saline (VBS, 1.8 mM Na-barbital, 3.3 mM barbituric acid, 147 mM NaCl, pH 7.5) or Tris-based solutions were used as assay buffers. 0.1% gelatin – VBS (GVBS) or hypotonic 1/3 GVBS was used in ^125^I-C4bp binding assays.

### Bacteria, plasmids and growth conditions

The bacterial strains and plasmids used in this study are listed in Table 1. For the C4bp-binding and inhibition assays as well as for the serum adsorption assay, bacteria were grown in the RPMI 1640 medium at 37°C. This medium increases YadA expression. Prior to use, exponential-phase bacteria were washed with VBS or PBS. When appropriate, antibiotics were added to the growth medium at the following concentrations: kanamycin (Km), 100 µg/ml in agar plates and 20 µg/ml in broth, chloramphenicol (Clm), 20 µg/ml, and ampicillin, 50 µg/ml.

**Table 1:** Bacteria and plasmids used in this work.

Bacterial strains and plasmids	Description	Source or reference
*Y. enterocolitica* strains		
6471/76 (YeO3)	Serotype O:3, fecal isolate, wild type	[57]
31761	Serotype O:3, fecal isolate	HUSLAB
49008	Serotype O:3, blood isolate	HUSLAB
49491	Serotype O:3, blood isolate	HUSLAB
YeO3-028	Δ*yadA::*Km-GenBlock, Km^R^, derivative of YeO3	[30]
YeO3-028-R1	Spontaneous rough derivative of YeO3-028, Km^R^	[30]
YeO3-028-OCR	Spontaneous OC mutant derivative of YeO3-028-R1, Km^R^	[30]
YeO3-028-OC	Δ(*wzx-wbcQ*) derivative of YeO3-028, Km^R^	[30]
6471/76 –c (YeO3-c)	Virulence plasmid cured derivative of YeO3	[57]
YeO3-R1	Spontaneous rough derivative of YeO3-c	[38]
YeO3-c-OC	Δ(*wzx-wbcQ*) derivative of YeO3-c	[30]
YeO3-c-OCR	Spontaneous rough derivative of YeO3-c-OC	[30]
YeO3-c-Ail	Δ*ail::*Km-GenBlock, Km^R^, derivative of YeO3-c	[30]
YeO3-c-Ail-OC	Spontaneous OC mutant derivative of YeO3-c-Ail, Km^R^	[30]
YeO3-c-Ail-R	Spontaneous rough derivative of YeO3-c-Ail, Km^R^	[30]
YeO3-c-Ail-OCR	Spontaneous OC mutant derivative of YeO3-c-Ail-R, Km^R^	[30]
8081	Serotype O:8, wild type	[77]
YeO8-116	Δ*yadA*::Km-GenBlock, Km^R^, derivative of 8081	This work
90936	Serotype O:9, quality assessment strain	HUSLAB
34884	Serotype O:9, blood isolate	HUSLAB
27675	Biotype 1A, virulence plasmid negative, fecal isolate	HUSLAB
*E. coli* strains		
JM103	sequencing host strain	[78]
S17-1		[79]
JM109	*reaA1 Δlac-pro endA1 gyrA96 thi-1 hsdR17 supE44 relA1 F′ traD36 proAB^+^ lacI^q^ZΔM15*	[80]
C600	*thi thr leuB tonA lacy supE*	[81]
HB101/pRK2013	Triparental conjugation helper strain, Km^R^	[82]
Plasmids		
pTM100	Mobilizable derivative of pACYC184, Clm^R^	[83]
pTM100-ail	*ail* gene cloned as 1711 bp PCR fragment into EcoRV site of pTM100, Clm^R^	Biedzka-Sarek *et al.* unpublished
pYMS4450	promoterless *yadA* cloned into pL2.1; *yadA*, Amp^R^	[46]
pL2.1	pBR322 with the P_tac_ promoter, Amp^R^	[84]
pYV8081	Virulence plasmid of 8081	[77]
pYMS3221x	*yadA* cloned in 4.4 kb XbaI-PvuI fragment between EcoRV-XbaI sites of pTM100	This work
pYMS3223	Km-GenBlock inserted in the *Cla*I site of pYMS3221x	This work
pUC4K	Source of the Km-GenBlock cassette, Amp^R^ Km^R^	GE Healthcare Life Sciences, cat. no 27-4958-01

HUSLAB, Helsinki University Central Hospital, Helsinki, Finland.

### Construction of strains

To generate a YadA-negative strain of *Y. enterocolitica* O:8 the *yadA* -gene of pYV8081 was cloned in a 4.4 kb *Xba*I-*Pvu*I fragment (nt 45398–49838 of pYV8081, accession number NC_008791) between the *Eco*RV and *Xba*I sites of pTM100 to generate plasmid pYMS3221x. The kanamycin resistance GenBlock (KmGB) removed by *Acc*I digestion from pUC4K was cloned into the *Cla*I site within the *yadA* gene of pYMS3221x to obtain pYMS3223. The expression of YadA was abolished by the KmGB-insertion (data not shown). pYMS3223 was transformed into *E. coli* S17-1 and mobilized into the wild type *Y. enterocolitica* O:8 strain 8081 and recombinants, which had lost the vector plasmid due to double crossing-over between pYV8081 and pYMS3223, were screened for by looking for Km^R^ Clm^S^ strains and one such strain was named as YeO8-116. YeO8-116 did not express YadA as verified by SDS-PAGE and autoagglutination tests (data not shown). Restriction digestions and Southern hybridizations of the isolated virulence plasmid of YeO8-116 showed that it carried the KmGB and an inactivated *yadA* -gene. In addition, YeO8-116 was calcium dependent, and produced the Yop proteins as released proteins identical to the wild type strain (data not shown). The strain Ye08-116 has been used also in earlier studies [43]–[45]. Plasmid pTM100 was electroporated into the *E. coli* JM109 strain. Triparental conjugation was used to mobilize the plasmids pTM100 and pTM100-ail (from JM109/pTM100 and JM109/pTM100-ail) with a help of *E. coli* HB101/pRK2013 to YeO3-c-Ail-OCR. The matings were performed as described elsewhere [30].

### C4bp binding and inhibition assays

Bacteria grown to mid-logarithmic phase were suspended in hypotonic 1/3 GVBS. A 50 µl (3×10^8^) aliquot of the bacterial suspension was incubated with 50 µl of radiolabeled C4bp (5,000–20,000 cpm) for 30 minutes at 37°C. The assays using BSA (0–300 nM/assay), unlabeled C4bp (0–300 nM/assay) or factor H (0–300 nM/assay) as competitors were performed with 40 µl of bacteria (3×10^7^) in reaction mixtures containing ^125^I-C4bp, or ^125^I-BSA as a control. The effects of heparin (0–1000 µg/ml) and NaCl (50–650 mM) on binding were assayed in reaction mixtures containing ^125^I-C4bp and 40 µl of bacteria (3×10^8^). After incubation the mixtures were centrifuged through 20% (w/v) sucrose in 1/3 GVBS to pellet the bacteria with the bound radiolabeled protein. Tubes were frozen, the bottoms of the tubes were cut out and radioactivities in the pellets and supernatants were measured with a γ-counter (Wallac, Finland). The ratios of bound to total activities were calculated.

### Serum adsorption assay

Bacteria (3×10^8^) were incubated in 5% heat-inactivated serum at 37°C for 30 min. Thereafter, the bacteria were washed 5–6 times with 400 µl of PBS or 1/3 PBS and bound proteins were eluted with 0.1 M glycine-HCl (pH 2.7) or PBS, respectively. Supernatants were collected and those acidified due to elution with glycine were additionally neutralized with 1 M Tris-HCl (pH 7.5). Samples of the last wash and elution fractions were subjected to a non-reducing 8% SDS-PAGE gel electrophoresis and subsequently transferred onto nitrocellulose membranes. The membranes were blocked and incubated with 1∶7,500 diluted sheep anti-human C4bp antiserum (The Binding Site, Birmingham, UK) and further with 1∶10,000 diluted peroxidase-conjugated donkey anti-sheep antiserum (Jackson Immunoresearch). The proteins were detected by enhanced chemiluminescence.

### Cofactor assay for C4b inactivation

The cofactor assay was performed to analyze the effect of C4bp on FI-mediated cleavage of C4b. Bacteria (4×10^9^) were incubated with C4bp (final concentration 50 µg/ml) in 40 µl of PBS for 30 min at 37°C with shaking. After washing for four times with PBS, bacteria (10^9^) were pelleted and resuspended in 30 µl of PBS containing FI (final concentration 50 µg/ml) and C4b (final concentration 35 µg/ml). The reactions were incubated for 45 min at 37°C with shaking. A reaction where C4b and FI were incubated for 45 min at 37°C with 50 µg/ml of C4bp was used a positive control. In addition, a negative control comprising of C4b and FI incubated without C4bp, was included. After incubation the samples were centrifuged, supernatants were collected, mixed with Laemmli buffer and subjected to 12.5% SDS-PAGE and immunoblotting using rabbit anti-human C4c antiserum (DAKO, 1∶5,000). The bound anti-human C4c antibodies were detected using HRP-conjugated swine anti-rabbit IgG (DAKO; 1∶5,000). Supernatants were also analyzed by immunoblotting with sheep anti-human C4bp antiserum to exclude unbound C4bp.

### Purification of YadA using Triton X-114 (Tx-114)

The extracts Tx-YadA (from *E. coli* JM103/pYMS4450), Tx-Ail (from *E. coli* JM109/pTM100-ail) and vector control extracts from *E. coli* strains JM103/pL2.1 and JM109/pTM100 were prepared as described previously [46] with slight modifications. Briefly, bacteria were grown overnight at 37°C in 400 ml of Luria broth supplemented with appropriate antibiotics. Bacteria were centrifuged (3,000×*g*, 15 min) and incubated on ice for 1 h in 20 ml of lysis buffer (10 mM EDTA, 50 mM glucose, 25 mM Tris-HCl [pH 8.0], 5 mg/ml of lysozyme). Triton X-114, prepared as described previously [47], was then added to the lysate to a final concentration of 5%. The extraction was carried out by incubating the mixture at 4°C for 24 h with slow rocking. Subsequently, the mixture was incubated overnight at 37°C to separate the water and Tx-114 phases followed by centrifugation (4,000×*g*, 10 min) to clear the phases. The Tx-114 phase was recovered and stored at 4°C.

### Purification of Ail using b-octylglucoside (OG)

The OG-Ail and vector control extracts from *E. coli* JM109/pTM100-ail and JM109/pTM100 were prepared using OG as described elsewhere [37].

### C4bp ligand blotting

Samples from Tx-YadA, Tx-Ail and OG-Ail as well as from the three control extracts (see above) were run into a 7.5–17.5% SDS-PAGE gel. Proteins were electrotransferred onto a nitrocellulose membrane. Membranes were blocked with 5% skimmed milk and incubated with the radioactively labeled C4bp (10^6^ cpm/assay) or 5% NHS. Binding of the protein was detected by autoradiography or immunoblotting with rabbit anti-human C4bp (5 µg/ml) as described above. YadA on the membrane was detected by immunoblotting with the monoclonal antibody 3G12 [48].

### Accession numbers

The GenBank (http://www.ncbi.nlm.nih.gov/Genbank/) accession number for the gene sequence of Ye O:8 *YadA* is X13881. For the protein sequences the accession numbers are CAE53849 for Ail (Ye serotype O:3) and CAA32086 for YadA (Ye 6471/76 serotype O:3).

## Results

### *Y. enterocolitica* binds complement regulator C4bp

**Figure 1:**
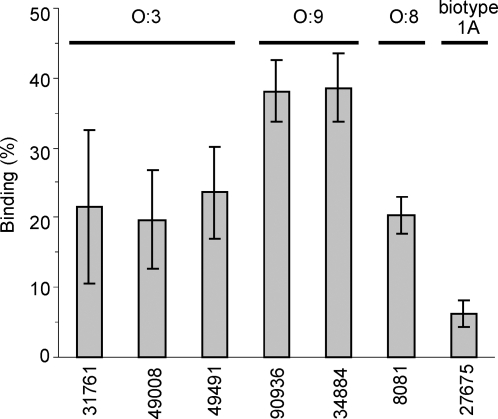
Binding of ^125^I-C4bp to *Y. enterocolitica* serotypes O:3, O:8, O:9 and biotype 1A. Indicated strains were incubated with ^125^I-C4bp for 30 min at 37°C. Subsequently, they were centrifuged through 20% sucrose to separate bacteria-bound ^125^I-C4bp from the unbound protein. Radioactivity was measured with a γ-counter. The binding of ^125^I-C4bp is expressed as percentage of the total radioactivity. Mean±SD values from two experiments performed in duplicate are shown.

In order to study whether *Y. enterocolitica* binds the classical and lectin pathway complement inhibitor C4bp serotype O:3, O:8, O:9 and biotype 1A strains were incubated with ^125^I-C4bp and phase-separated from unbound ^125^I-C4bp by centrifugation through 20% sucrose. As shown in Fig. 1, all strains belonging to pathogenic pYV-positive serotypes O:3, O:8 and O:9 bound ^125^I-C4bp with a binding capacity of ∼20–40%. The less virulent pYV-negative biotype 1A *Y. enterocolitica* strain (27675) bound ^125^I-C4bp significantly less or not at all. The binding percentages by strains that did not bind C4bp (including *E. coli* K12 C600) ranged between 1–8% under the conditions used (not shown).

### Cofactor activity of *Y. enterocolitica*–bound C4bp

**Figure 2:**
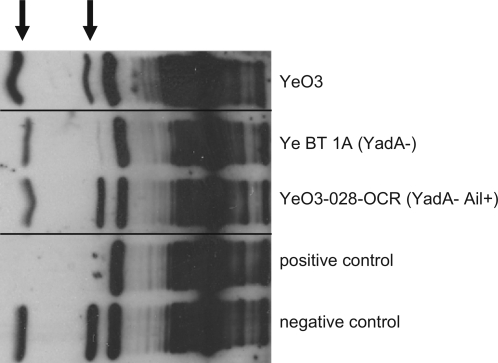
Cofactor activity of *Y. enterocolitica*-bound C4bp for C4b cleavage. *Y. enterocolitica* serotype O:3 wild-type bacteria (YeO3), YadA-negative less virulent biotype 1A strain 27675 (BT 1A) and Ail-expressing strain YeO3-028-OCR were preincubated with C4bp and after extensive washings exposed to factor I and C4b. C4b and its cleavage products from the supernatants were detected using polyclonal antibodies against C4c. As positive and negative controls, assays containing C4b and FI with or without C4bp, respectively, were included. Inactivation of C4b is demonstrated by the appearance of C4b cleavage fragments (indicated with arrows).

To examine cofactor activity of the *Y. enterocolitica*–bound C4bp, serotype O:3 wild type bacteria and Ail-expressing YeO3-028-OCR strain were incubated with purified C4bp as described in Experimental procedures. A clinical isolate, less virulent Ye biotype 1A strain was included as a control because the strain bound C4bp clearly less than the other two strains used. Unbound C4bp was removed by extensive washing with PBS. The bacteria with surface-bound C4bp were subsequently incubated with FI and C4b. Following the incubation, the C4bp-cofactor activity was verified by immunodetection of C4b-cleavage products in the supernatants using anti-C4c antibodies. As a polyclonal antibody the anti-C4c antibody detects the α′-chain, the β-chain and cleavage fragments of the α′-chain. As shown in Fig. 2, *Y. enterocolitica*-bound C4bp displayed cofactor activity for FI-mediated cleavage of C4b with both strains YeO3 and Ye-028-OCR. This is indicated by the appearance of the C4b α′-chain cleavage fragments (Fig. 2). The molecular weight of the cleavage fragment generated by *Y. enterocolitica*-bound C4bp corresponds to the fragment generated in the absence of bacteria by FI incubated with C4bp and C4b (Fig. 2, positive control lane). Clearly less cleavage products were seen with the biotype 1A strain, as expected based on the low binding of radiolabeled C4bp. No cleavage was observed when bacteria were incubated with FI and C4b suggesting that C4bp bound to bacterial surface is essential for the observed C4b cleavage (data not shown).

### Identification of C4bp receptors on *Y. enterocolitica*

**Figure 3:**
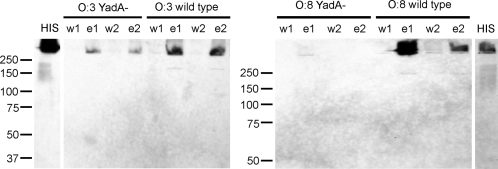
Binding of serum C4bp to the wild type and YadA-negative *Y. enterocolitica* serotypes O:3 and O:8. Wild type bacteria (Ye03 and 8081) and their YadA-negative derivatives (YeO3-028 and YeO8-116, respectively) (3×10^8^) were incubated in 5% heat-inactivated serum (HIS) for 30 min. Bacteria washed in 1/3 PBS were subjected to elution with PBS while those washed with PBS were subjected to elution with 0.1 M glycine-HCl (pH 2.7). The 1/3 PBS or PBS wash fractions (w1 and w2, respectively) and PBS or 0.1 M glycine-HCl (pH 2.7) elute fractions (e1 and e2, respectively) were separated by 8% non-reducing SDS-PAGE and analyzed by immunoblotting using a sheep anti human-C4bp antiserum.

Serum resistance of *Y. enterocolitica* depends greatly on the expression of the pYV-encoded YadA protein [29]–[31],[49],[50]. Thus, we first examined the role of YadA in the acquisition of serum C4bp. To this end we incubated the wild type O:3 and O:8 strains (YeO3 and 8081, Table 1) and their YadA-negative pYV-positive derivatives (YeO3-028 and YeO8-116, respectively) in heat-inactivated serum (HIS). After extensive washings, the bacteria-bound serum proteins were eluted and subjected to immunoblotting using anti-C4bp antiserum. Material eluted from the wild type O:3 and O:8 strains contained C4bp while strains lacking YadA displayed significantly less C4bp in the eluted fractions (Fig. 3). This suggested that YadA is involved in C4bp binding. Residual C4bp-binding by YadA-negative strains, however, suggested a role for other *Y. enterocolitica* factor(s) in C4bp-binding.

**Figure 4:**
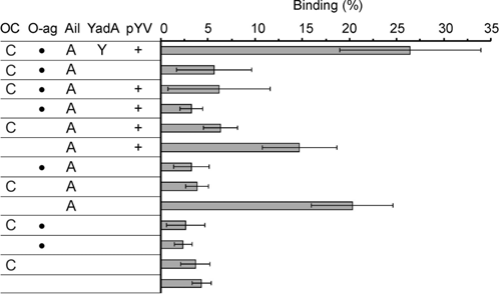
Binding of ^125^I-C4bp to *Y. enterocolitica* serotype O:3 strains. The bacteria were incubated with ^125^I-C4bp as described in Fig. 1. The factors expressed by Ye O:3 strains are marked as follows: YadA, Y; Ail, A, O-ag,•; OC, C. Strains carrying the pYV plasmid are marked with +.

**Figure 5:**
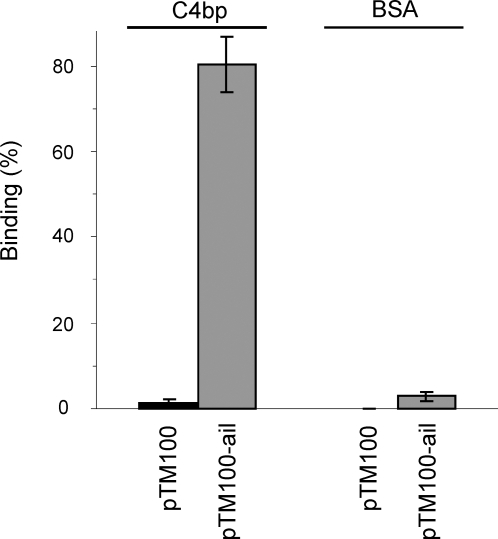
Binding of ^125^I-C4bp to Ail. The strain YeO3-c-Ail-OCR was complemented in *trans* with pTM100-ail expressing the cloned *ail* gene (YeO3-c-Ail-OCR/pTM100-ail). YeO3-c-Ail-OCR/pTM100 was used as the vector control. The bacteria were incubated with ^125^I-C4bp or ^125^I-BSA as described in Fig. 1.

To identify C4bp receptors on *Y. enterocolitica* O:3 surface a set of 12 strains expressing YadA, Ail, LPS O-ag and OC in different combinations was tested for the ability to bind ^125^I-C4bp in 1/3 GVBS buffer containing 50 mM NaCl. YadA was indispensable for the maximal C4bp binding. Both pYV- and YadA-negative strains (YeO3-c and YeO3-028, respectively) displayed equally low levels of bound C4bp suggesting that the main factor responsible for the binding is pYV-encoded YadA (Fig. 4). Almost all YadA-negative strains bound much less C4bp than the wild type strain. The two sole exceptions were strains expressing Ail in the absence of both O-ag and OC (Fig. 4, YeO3-c-OCR and YeO3-028-OCR). Both Ail-expressing strains were found to bind ^125^I-C4bp. The fact that the removal of either O-ag (Fig. 4, YeO3-028-R, YeO3-R1) or OC (Fig. 5, YeO3-028-OC, YeO3-c-OC) was not sufficient to promote Ail-C4bp interaction suggests that either of them can block Ail-mediated C4bp-binding to the bacterial surface. To confirm Ail-mediated C4bp binding, we complemented in *trans* the YeO3-c-Ail-OCR strain (YadA^−^, Ail^−^, O-ag^−^, OC^−^) with pTM100-ail carrying the cloned *ail* gene. The resulting strain YeO3-c-Ail-OCR/pTM100-ail restored C4bp-binding ability, while the vector control YeO3-c-Ail-OCR/pTM100 strain was unable to bind the CP-regulator (Fig. 5). C4bp binding was shown to be specific since no binding of ^125^I-labeled control protein, BSA, to any of the tested strains was detected (Fig. 5). In addition, the strain with *trans*-complemented *ail* (YeO3-c-Ail-OCR/pTM100-ail) displayed about four-fold higher C4bp binding capacity (80%) than the YeO3-c-OCR strain expressing Ail from the chromosomally-located *ail* gene (Figs. 4 and 5). This difference, however, can be explained by the overexpression of Ail by pTM100-ail due to a copy-number effect (data not shown).

Neither O-ag nor OC contributed to C4bp binding since YadA- Ail- negative strains expressing full LPS (YeO3-c-Ail) or its rough (YeO3-c-Ail-R) or OC-less (YeO3-c-Ail-OC) derivatives bound only negligible amounts of C4bp (Fig. 4).

**Figure 6:**
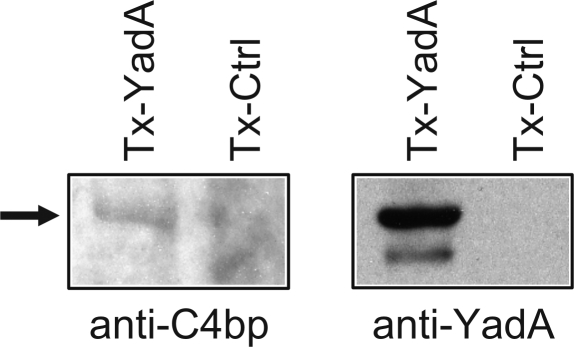
Affinity blotting analysis of C4bp-binding to Triton X-114-extracted YadA. Membrane proteins were extracted from *E. coli* JM103/pYMS4450 expressing YadA of *Y. enterocolitica* serotype O:3 (Tx-YadA) and *E. coli* JM103/pL2.1 carrying the empty vector (Tx-Ctrl). Extracts were subjected to SDS-PAGE and proteins were transferred onto nitrocellulose membrane. The membrane was blocked in 5% skimmed milk in PBS and incubated with 5% normal human serum. After washing C4bp-bound to extracted proteins was detected using rabbit anti-human C4bp antibody (anti-C4bp). The YadA-bound C4bp is indicated with an arrow. In parallel, YadA protein on the membrane was detected by the monoclonal antibody 3G12 (anti-YadA).

To see a direct interaction of YadA and Ail with C4bp we tested the binding of serum C4bp to TritonX-114 extracted YadA and Ail, Tx-YadA and Tx-Ail, respectively, in affinity blotting. C4bp binding to YadA trimer was observed (Fig. 6). Binding to Tx-Ail, however, could not be detected (data not shown). Also β-octylglucoside-extracted Ail (OG-Ail) failed to bind C4bp when tested in a ligand blotting assay (data not shown). This suggests that Ail loses its appropriate conformational structure upon extraction and/or processing for SDS-PAGE.

### Effect of salt, heparin and factor H on C4bp binding to *Y. enterocolitica*

**Figure 7:**
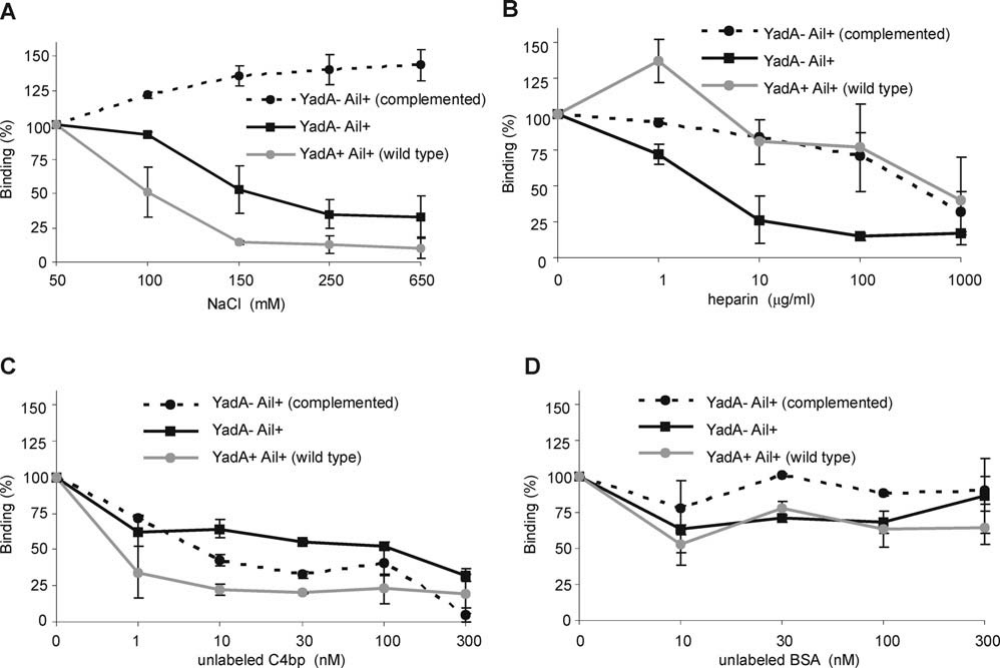
The effect of NaCl (A), heparin (B), unlabeled C4bp (C) or BSA (D) on binding of ^125^I-C4bp to *Y. enterocolitica*. Wild type (YadA+ Ail+, YeO3) and YadA-negative (YadA- Ail+, YeO3-028-OCR and YadA- Ail+ (complemented), YeO3-c-Ail-OCR/pTM100-ail) bacteria were incubated with ^125^I-C4bp as described in Fig. 1. The binding is presented as percentage of the ^125^I-C4bp binding occurring in the presence of 50 mM NaCl without any additives. In the wild type strain Ail protein is masked by O-ag and OC and thus the binding represents properties of the YadA-C4bp interaction.

Electrostatic forces and ion pairings have been suggested to be essential for C4bp binding to C4b [51]. The nature of C4bp binding to YadA and Ail was examined using the wild type strain YeO3 that expresses both YadA and Ail (but the latter is blocked by O-ag and OC as demonstrated above), mutant strain YeO3-028-OCR that expresses unblocked Ail, and YeO3-c-Ail-OCR/pTM100-ail expressing Ail in *trans*. Bacteria were incubated with ^125^I-C4bp in 1/3 GVBS alone or supplemented with NaCl to create a salt-concentration gradient ranging from 50 to 650 mM. After centrifugation through 20% sucrose bound ^125^I-C4bp was measured using a gamma-counter. In general, at low salt concentrations both YadA- and Ail-mediated C4bp-binding was the highest and showed a tendency to decrease with increasing salt concentrations (Fig. 7A). The C4bp binding to YeO3-c-Ail-OCR/pTM100-ail was not affected by an increase in salt concentration. YadA-mediated C4bp-binding, however, was more salt sensitive than Ail-mediated binding. A two-fold decrease in C4bp binding to YadA (strain YeO3) was observed already at NaCl concentration of 100 mM while Ail-mediated C4bp-binding at this salt concentration was not affected. Thus, C4bp binding to YadA depends on ionic interactions between the proteins. The fact that C4bp-binding to YadA, similarly as that to C4b [51], was almost completely abolished in the presence of 250 mM salt (Fig. 7A), suggests that YadA and C4b have affinity for C4bp at site(s) with similar properties.

Heparin binds to the N-termini of C4bp α-chains, i.e. to CCP1-3 [52]–[54]. We tested whether heparin inhibits the binding of C4bp to YadA- or Ail-expressing strains (Fig. 7B). Bacteria were incubated with ^125^I-C4bp in the presence of heparin (0–1000 µg/ml). As shown in Fig. 7B heparin efficiently and dose-dependently inhibited ^125^I-C4bp binding to Ail, while significant reduction of ^125^I-C4bp binding to YadA could only be observed at the highest heparin concentration of 1000 µg/ml. Delayed response to heparin was observed with the strain over-expressing Ail (YeO3-c-Ail-OCR/pTM100-ail).

The relative binding of ^125^I-C4bp to Ail-expressing strain Ye-028-OCR could be dose-dependently reduced to 55% by adding 0–300 nM factor H. C4bp binding to YeO8 wt strain was not affected by the addition of 0–300 nM factor H (data not shown).

### Specificity of C4bp binding to *Yersinia*

To verify whether YadA- and Ail-mediated ^125^I-C4bp-binding was specific, wild type and YeO3-028-OCR strains were incubated with ^125^I-C4bp in the presence of unlabeled C4bp or BSA (0–300 nM). In this assay, ten-fold less bacteria (3×10^7^) were used when compared to the heparin and salt inhibition experiments. Under these conditions ^125^I-C4bp binding to both, YadA (wild type) and Ail (YeO3-028-OCR and YeO3-c-Ail-OCR/pTM100-ail), was significantly inhibited in the presence of unlabeled C4bp (Fig. 7C). YadA-mediated ^125^I-C4bp-binding was inhibited to a greater extent than that of Ail. The presence of BSA did not affect the ^125^I-C4bp binding neither to YadA nor to Ail (Fig. 7D). With ten-fold higher numbers of bacteria, the inhibition with unlabeled C4bp could not be observed possibly because of excessive amounts of C4bp-binding surface proteins, YadA and Ail (data not shown).

## Discussion

The complement system is an essential part of host defense against many microorganisms. A number of pathogens, however, have evolved mechanisms to subvert complement activation at different steps of the cascade. To survive and establish infection in the gut and surrounding tissues, *Y. enterocolitica* must resist complement-mediated opsonization and lysis. It is thus equipped with surface factors that confer resistance to serum, such as the outer membrane proteins, YadA and Ail [29]–[33],[55]. Although it has been shown that Ail promotes resistance to complement killing, the mechanism of Ail-mediated serum resistance has remained unknown. YadA, in turn, has been shown to be the major serum resistance determinant of *Y. enterocolitica* [29],[30],[49]. Thus, not surprisingly, mechanisms underlying YadA-mediated resistance have for long been of interest. It has been speculated that the formation of YadA-composed velvet-like coat on the bacterial surface could by itself act as a shield protecting against complement [56],[57]. There is evidence, however, for YadA-mediated binding of the alternative pathway regulator FH and inhibition of the complement cascade at both C3 and C9 levels [40],[58]. This manifests as a reduced binding of C3b and as a failure of the membrane attack complex to incorporate into the outer membrane of *Y. enterocolitica* [49]. Lipopolysaccharide O-ag and OC are involved in complement-resistance indirectly [30]. They block outer membrane proteins, such as small-sized Ail, thereby having a negative influence on bacterial resistance to serum [30].

This study demonstrated a novel immune evasion mechanism of *Y. enterocolitica*, C4bp binding by YadA and Ail proteins. All serotypes tested, O:3, O:8 and O:9, were shown to bind the host complement regulator C4bp to avoid opsonophagocytosis and bactericidal action of serum (Fig. 1). The bacteria also acquired this CP-inhibitor from serum, as demonstrated by serum adsorption assays (Fig. 3). In addition, binding of purified radiolabeled C4bp to *Y. enterocolitica* could be observed (Figs. 1 and 4). This shows that the binding is direct and does not involve other serum proteins. Importantly, FI cofactor assay showed that C4bp bound to *Y. enterocolitica* surface was functionally active (Fig. 2). By binding C4bp *Y. enterocolitica* can thus inhibit antibody-mediated CP, and the LP.

C4bp receptors on *Y. enterocolitica* surface were identified using a set of serotype O:3 mutants expressing YadA, Ail, O-ag and OC in different combinations (Fig. 4). Analyses of ^125^I-labeled C4bp binding to *Y. enterocolitica* O:3 strains showed that YadA was crucial for capturing this CP regulator. Therefore, YadA- or pYV-negative mutants bound only marginal amounts of C4bp, exceptions being the strains expressing Ail in the absence of O-ag and OC (Fig. 4). Thus, Ail could bind C4bp solely when accessible on the outer membrane. This was additionally confirmed by *trans*-complementing *ail* in a strain missing all four factors (YeO3-c-Ail-OCR). Since this strain lacks O-ag and OC, C4bp binding to Ail was strongly favored (Fig. 5). The demonstration of C4bp receptors as YadA and Ail correlated with the previously published serum resistance results of these Ye O:3 strains [30]. These results revealed that the major serum resistance determinant of *Y. enterocolitica* was YadA and that the removal of LPS O-ag and OC potentiated Ail-mediated complement resistance of YadA-negative strains [30]. It is possible that during infection the production of LPS, O-ag, and OC is suppressed. Similar phenomenon has been observed for Salmonella [59],[60]. In addition, LPS was shown not to contribute to serum resistance directly. Accordingly, in the present work we observed binding of C4bp neither to O-ag nor to OC (Fig. 4).

YadA is a member of a large family of surface proteins of Gram-negative bacteria. These trimeric autotransporter proteins exert many functions and are required for full virulence of pathogenic species. Some of these proteins, such as *Actinobacillus actinomyctemcomitans* Omp100, *E. coli* EibD, *Haemophilus ducreyi* DsrA and *Moraxella catarrhalis* UspA1 and UspA2, confer resistance to serum [14], [61]–[66]. Interestingly, DsrA, UspA1 and UspA2 have been shown to capture C4bp [14],[63]. Apparently, C4bp-binding is a mechanism shared by multiple members of this family of autotransporters. The interaction between YadA and C4bp appeared to be ionic strength-dependent (Fig. 7A). Interestingly, salt inhibited YadA-C4bp interaction similarly to that between C4bp and C4b [51]. The fact that the positively charged cluster of amino acids between CCP1 and CCP2 is involved in C4b binding [54] would suggest that these CCPs are needed also for the YadA-C4bp interaction. Heparin inhibition assay, however, showed discordant results (Fig. 7B). The electronegative polysaccharide, heparin, alike C4b, binds to CCP1-2, and thus partially competes with C4b for C4bp binding [52]–[54]. Heparin inhibition data showed an initial increase in C4bp binding to YadA at low heparin concentrations (Fig. 7B). This could be theoretically explained by the binding of C4bp oligomers, formed in the presence of heparin, to YadA. Higher doses of heparin inhibited rather weakly C4bp binding to YadA, and 50% inhibition of the binding could only be observed at the highest heparin concentration of 1 mg/ml (Fig. 7B). Thus, the C4bp binding sites for C4b and YadA do not seem to be identical, but most likely are overlapping. Electrostatic forces thus mediate the YadA-C4bp complex formation.

Ail belongs to a family of β-barrel outer membrane proteins that include *Salmonella enterica* serovar Typhimurium PagC and Rck, and *Enterobacter cloacae* OmpX [67]–[71]. These proteins, though highly similar in structure, do not appear to share many of their functions. The only protein sharing serum resistance phenotype with Ail is Rck [72]. Here we provided evidence for Ail-mediated C4bp binding. The mechanism of C4bp binding to Ail appeared to be different from that of C4b and YadA, as heparin efficiently and dose-dependently inhibited the binding of C4bp to Ail (Fig. 7B). This observation suggested that Ail-binding involved the CCP1-3 domains of C4bp α-chain. The Ail-C4bp interaction was also less sensitive to salt when compared to that of YadA-C4bp (Fig. 7A) or C4b-C4bp interactions [51]. Thus, other than electrostatic forces, e.g. hydrophobicity, could also be involved in this interaction. Ail binding sites on C4bp, however, are most likely not fully equivalent, though possibly overlapping, with those involved in C4b-C4bp or YadA-C4bp interactions.

*Y. pestis*, the causative agent of plague, has been shown to be resistant against complement-mediated killing and to bind C4bp [25]. In contrast to *Y. enterocolitica*, the surface protein Ail (also called OmpX) seems to be solely responsible for the serum resistance property in *Y. pestis* [73],[74]. *Y. pestis* Ail protein shares about 70% sequence identity with *Y. enterocolitica* Ail. As a result of several gene mutations, however, *Y. pestis* does not express YadA or O-ag [75]. Here we have shown that in YadA-negative *Y. enterocolitica* strain, removing the Ail-masking O-ag and OC greatly enhances the binding of C4bp (Fig. 4). Based on these previous findings and our current results about Ail binding C4bp, it is probable that constitutively expressed Ail binds C4bp also on the surface of *Y. pestis* and is, at least partly, responsible for the high complement resistance.

**Figure 8:**
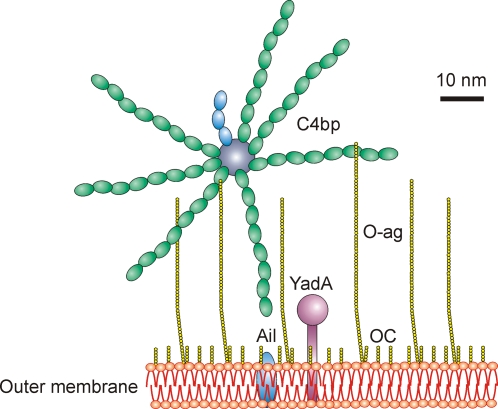
Schematic model of C4bp binding to *Y. enterocolitica* surface structures. *Y. enterocolitica* expresses outer membrane proteins, YadA and Ail, that are able to capture C4bp. Ail, however, is masked by LPS O-ag chains and OC branches. Hexasaccharide OC is abundant on the *Y. enterocolitica* surface and forms a branched structure with O-ag. Removal of the O-ag and OC can unveil Ail and promotes Ail-mediated C4bp binding. C4bp [59],[60], YadA [76], Ail, outer membrane and O-antigen (1 nm per sugar residue) are drawn to scale.

In summary, this study provides the first evidence that *Y. enterocolitica* acquires the CP regulator C4bp in a functionally active form able to promote degradation of C4b. The binding depends on the two outer membrane proteins YadA and Ail, the latter binding C4bp only when well surface-exposed, i.e., not blocked by O-ag or OC (Fig. 8). *Y. enterocolitica* is thus able to take advantage of the captured C4bp and is likely to be able to prevent both C4b-mediated opsonization and formation of the CP C3-convertase (C4bC2a). Consequently, the bacteria can avoid complement-mediated lysis and increase their chances to survive in the human host. It is also remarkable that *Y. enterocolitica* uses both YadA and Ail to recruit both the AP and CP regulators FH and C4bp, respectively. This way the pathogen can ascertain that it will be protected from complement activation during different phases of infection.
